# Supplementary material for: Thermal stress responses of Sodalis glossinidius, an indigenous bacterial symbiont of hematophagous tsetse flies
Source: PLoS Negl Trop Dis. 2019 Nov 18;13(11):e0007464. doi: 10.1371/journal.pntd.0007464 (PMC6887450; doi:10.1371/journal.pntd.0007464)

Fig. S1

A

***dnaK* promoter**

5′CTATTACCC**CTTGA**TGCAGGGCAGCATGA**CCCCATCT**T
ATTTGCATCCAACGTTGTTAAACCTGGGAAATTATCGGGC
GGCGAAAACTTGAAAAGGTAATTTTCGGCCGCATATCAGG
TTTACGACCACAACGAAATTGAACTTTAGT***GGAGAT***GTTT
AG**ATG**

***grpE* promoter**

5′TCATTATGCGTCATCC**CTTGAA**TCCCCGCTATTCAT**CC**
CCATAATAACCGGACGAGCGAGAATAATGCTGAACCGC***GG***
AGAAAACTCATG**ATG**

B

σ^{32} consensus sequence

CTTGAAN$_{(13-17)}$CCCCATNT

C

***dnaK-dnaJ* intergenic region**

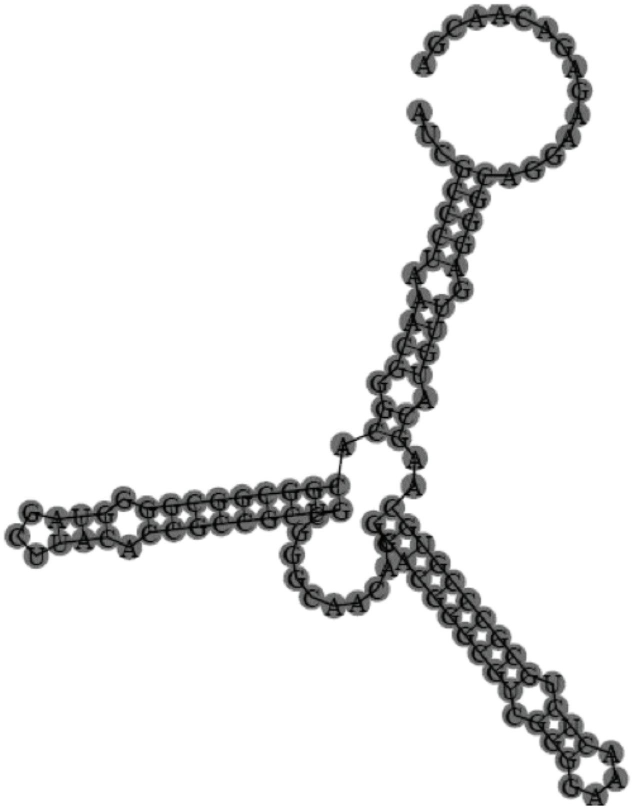

Supplement: S1 Fig — (A) Putative Sodalis promoters for the polycistronic dnaK and dnaJ mRNA and for monocistronic grpE mRNA are shown, based on homology to their E. coli promoters. Start codons are bolded, the Shine-Delgarno sequence is bolded and italicized, and the σ32 binding sites are bolded and underlined. (B) The consensus sequence for the σ32 binding site for E. coli [72]. (C) A potential secondary structure of the RNA corresponding to the dnaK–dnaJ intergenic region, generated using RNAfold from the ViennaRNA package [73, 74]. (PDF) [file pntd.0007464.s001.pdf]
