# Supplementary material for: Thermal stress responses of Sodalis glossinidius, an indigenous bacterial symbiont of hematophagous tsetse flies
Source: PLoS Negl Trop Dis. 2019 Nov 18;13(11):e0007464. doi: 10.1371/journal.pntd.0007464 (PMC6887450; doi:10.1371/journal.pntd.0007464)
Supplement: S2 Fig — Alignment of Sodalis DnaK, DnaJ, and GrpE with homologues from Escherichia coli MG1665 using Clustal Omega (https://www.ebi.ac.uk/Tools/msa/clustalo/). An asterisk (*) indicates positions that have a single, fully conserved residue. A colon (:) indicates conservation between groups that exhibit strongly similar properties, roughly equivalent to scoring > 0.5 in the Gonnet PAM 250 matrix. A period (.) indicates conservation between groups that exhibit weakly similar properties, roughly equivalent to scoring ≤ 0.5 and > 0 in the Gonnet PAM 250 matrix. For DnaK, the boxed residues indicate a glycine (G) that interacts with GrpE, a glutamine (Q) that binds the unfolded protein substrate and an alanine (A) that is involved in synergistic activation of ATPase by DnaJ [75–77]. The overlined residues indicate DnaK amino acids predicted to interact with Mg-ADP [76, 78, 79]. The dashed underline indicates a motif found in DnaK from all gram-negative bacteria that is thought to be essential for ATP-dependent cooperative function with DnaJ and GrpE [80]. The threonine (T) with the dot is required for ATPase activity [81]. For DnaJ, the bracketed residues are conserved residues in the J-domain that interact with DnaK [82, 83]. The underlined residues are zinc-binding motifs that are predicted to bind the unfolded protein substrate [84–86]. The G/F region, which may modulate unfolded substrate binding to DnaK, is boxed, and the DIF motifs within this G/F region, which are involved in regulation of chaperone cycling by modulating a step after ATP hydrolysis [87, 88], are overlined. (PDF) [file pntd.0007464.s002.pdf]

Fig. S2

A

DnaK

```
Sodalis  MGKIIGIDLGTTNSCIAIIEGSKPRVLENSEGDRTTPSIIAYTQDGEILVGQPAKRQSVT  60
E. coli  MGKIIGIDLGTTNSCVAIMDGTTPRVLENAEGDRTTPSIIAYTQDGETLVGQPAKRQAVT  60
         ***************:**::*:.******:****************** ********:**

Sodalis  NPQNTLFAIKRLIGRRYQDEEVQRDVSIMPYKIVAADNGDAWLEVKGQKMAPPQISAEIL  120
E. coli  NPQNTLFAIKRLIGRRFQDEEVQRDVSIMPFKIIAADNGDAWVEVKGQKMAPPQISAEVL  120
         ****************:*************:**:********:***************:*

Sodalis  KKMKKTAEDYLGEPVTEAVITVPAYFNDTQRQATKDAGRIAGLDVKRIINEPTAAALAYG  180
E. coli  KKMKKTAEDYLGEPVTEAVITVPAYFNDAQRQATKDAGRIAGLEVKRIINEPTAAALAYG  180
         ****************************:**************:****************

Sodalis  LDKETGNRTIAVYDLGGGTFDISIIEIDDVDGEKTFEVLATNGDTHLGGEDFDSRLINYL  240
E. coli  LDKGTGNRTIAVYDLGGGTFDISIIEIDEVDGEKTFEVLATNGDTHLGGEDFDSRLINYL  240
         *** ************************:*******************************

Sodalis  VDEFKKDQGIDLRNDPLAMQRLKEAAEKAKIELSSAQQTDVNLPYITADGSGPKHMNLKV  300
E. coli  VEEFKKDQGIDLRNDPLAMQRLKEAAEKAKIELSSAQQTDVNLPYITADATGPKHMNIKV  300
         *:***********************************************.:******:**

Sodalis  TRAKLESLVEELVNRTLEPLKVALKDAGLSVSDIKDVILVGGQTRMPLVQKKVTDFFGKE  360
E. coli  TRAKLESLVEDLVNRSIEPLKVALQDAGLSVSDIDDVILVGGQTRMPMVQKKVAEFFGKE  360
         **********:****::*******:*********.************:*****::*****

Sodalis  PRKDVNPDEAVAIGAAVQGGVLAGDVKDVLLLDVTPLSLGIETMGGVMTPLIAKNTTIPT  420
E. coli  PRKDVNPDEAVAIGAAVQGGVLTGDVKDVLLLDVTPLSLGIETMGGVMTTLIAKNTTIPT  420
         **********************:*************************** **********

Sodalis  KHSQVFSTAEDNQSAVTIHVLQGERKRSGDNKSLGQFNLDGISPAMRGTPQIEVTFDIDA  480
E. coli  KHSQVFSTAEDNQSAVTIHVLQGERKRAADNKSLGQFNLDGINPAPRGMPQIEVTFDIDA  480
         ***************************:.*************.** ** ***********

Sodalis  DGILHVSAKDKNSGREQKITIKASSGLNEEEIQKMVQEAEANAESDRKFEALVQTRNQAD  540
E. coli  DGILHVSAKDKNSGKEQKITIKASSGLNEDEIQKMVRDAEANAEADRKFEELVQTRNQGD  540
         **************:**************:******::******:***** *******.*

Sodalis  HLLHSTRKQLEDAGDKLPADDKTAIEDALKNLDTVLKGEDKADIEAKMQALIQVSGKLLE  600
E. coli  HLLHSTRKQVEEAGDKLPADDKTAIESALTALETALKGEDKAAIEAKMQELAQVSQKLME  600
         *********:*:**************.**. *:*.******* ****** * *** **:*

Sodalis  VAQQQAQAAGDGGADGS--AKADDDVVDAEFEEVKDKK                      636
E. coli  IAQQQHAQQQTAGADASANNAKDDDVVDAEFEEVKDKK                      638
         :****      .***.*     ****************
```

B

DnaJ

```
Sodalis  MAKSDYYEILGVSRDAEEREIKKAYKRQAMKFHPDRNRGNAEAEAKFKEIKEAYEVLTDA  60
E. coli  MAKQDYYEILGVSKTAEEREIRKAYKRLAMKYHPDRNQGDKEAEAKFKEIKEAYEVLTDS  60
         ***.*********: ******:***** ***:*****:*: ******************:

Sodalis  QKRAAYDQYGHAAFEQGGMGGGGA-SGADFSDIFGDVFGDIFGGGR-RQRVSRGADLRYN  118
E. coli  QKRAAYDQYGHAAFEQGGMGGGGFGGGADFSDIFGDVFGDIFGGGRGRQRAARGADLRYN  120
         ***********************  .******************** ***.:********

Sodalis  MELSLEEAVRGVTREIRIPTLEECDVCHGSGAKPGTSAVTCPTCHGQGQVQMRQGFFAIQ  178
E. coli  MELTLEEAVRGVTKEIRIPTLEECDVCHGSGAKPGTQPQTCPTCHGSGQVQMRQGFFAVQ  180
         ***:*********:**********************.  *******.***********:*

Sodalis  QTCPTCQGQGKIIKDPCTKCHGHGRVEKSKTLSVKIPAGVDTGDRIRLSGEGEVGEHGAA  238
E. coli  QTCPHCQGRGTLIKDPCNKCHGHGRVERSKTLSVKIPAGVDTGDRIRLAGEGEAGEHGAP  240
         **** ***:*.:*****.*********:********************:****.*****

Sodalis  AGDLYVQVQVCKHPIFEREENNLYCEVPINFAMAALGGEIEVPTLDGRVKLKVPAETQTG  298
E. coli  AGDLYVQVQVKQHPIFEREGNNLYCEVPINFAMAALGGEIEVPTLDGRVKLKVPGETQTG  300
         ********** :******* **********************************.*****

Sodalis  KLFRMRGKGVKSVRGGSQGDLLCRVVVETPVKLNERQKQLLRELEESFGGPSGDQNSPRS  358
E. coli  KLFRMRGKGVKSVRGGAQGDLLCRVVVETPVGLNERQKQLLQELQESFGGPTGEHNSPRS  360
         ****************:************** *********:**:******:*::*****

Sodalis  KSFLDGVKKFFDDLTR                                            374
E. coli  KSFFDGVKKFFDDLTR                                            376
         ***:************
```

C

GrpE

```
Sodalis  MSSKEQNTPDEQVSQESEMEQGQQAEA--APETVDVVDPRDERIAELEAALSQAQQREHD  58
E. coli  MSSKEQKTPEGQAPEEIIMDQHEEIEAVEPEASAEQVDPRDEKVANLEAQLAEAQTRERD  60
         ******:**: *. :*  *:* :: **     :.: ******::*:*** *::** **:*

Sodalis  SVLRAKAEMENVRRRSEQDVEKAHKFALERFAGELLPVIDNLERALDMSDKANAELASTI  118
E. coli  GILRVKAEMENLRRRTELDIEKAHKFALEKFINELLPVIDSLDRALEVADKANPDMSAMV  120
         .:**.******:***:* *:*********:*  *******.*:***:::****  :::: :

Sodalis  EGIELTLKSLLDAVRKFGLDVVGDTHVPFNPEVHQAMTMLESDEHEPNQVMMVMQKGYTL  178
E. coli  EGIELTLKSMLDVVRK-GVEVIAETNVPLDPNVHQAIAMVESDDVAPGNVLGIMQKGYTL  179
         *********:**.*** *::*:.:*.**::*:****::*:***:  * :*: :*******

Sodalis  NGRLIRPAMVAVSKAKS                                           195
E. coli  NGRTIRAAMVTVAKAKA                                           196
         *** ** ***:*:***:
```
