# Supplementary material for: Thermal stress responses of Sodalis glossinidius, an indigenous bacterial symbiont of hematophagous tsetse flies
Source: PLoS Negl Trop Dis. 2019 Nov 18;13(11):e0007464. doi: 10.1371/journal.pntd.0007464 (PMC6887450; doi:10.1371/journal.pntd.0007464)
Supplement: S3 Fig — Alignment of Sodalis glossinidius DnaK with homologues from Escherichia coli MG1665 and the insect symbionts using Clustal Omaga (https://www.ebi.ac.uk/Tools/msa/clustalo/). The species corresponding to the protein accession numbers are as follows: WP_074011646.1, Candidatus Sodalis sp. SoCistrobi; KYP97672.1, Sodalis-like endosymbiont of Proechinophthirus fluctus; WP_025244843.1, Candidatus Sodalis pierantonius; WP_067565807.1, Candidatus Doolittlea endobia; WP_067567978.1, Candidatus Hoaglandella endobia; WP_014888228.1, secondary endosymbiont of Ctenarytaina eucalypti; WP_067497883.1, Candidatus Gullanella endobia; WP_067568929.1, Candidatus Mikella endobia; AIN47473.1, Candidatus Baumannia cicadellinicola; WP_014888738.1; secondary endosymbiont of Heteropsylla cubana; WP_083172452.1, secondary endosymbiont of Trabutina mannipara; WP_013975497.1, Candidatus Moranella endobia. An asterisk (*) indicates positions which have a single, fully conserved residue. A colon (:) indicates conservation between groups of strongly similar properties, roughly equivalent to scoring > 0.5 in the Gonnet PAM 250 matrix. A period (.) indicates conservation between groups of weakly similar properties, roughly equivalent to scoring = < 0.5 and > 0 in the Gonnet PAM 250 matrix. The boxed residues indicate a glycine (G) that interacts with GrpE, a glutamine (Q) that binds the unfolded protein substrate and an alanine (A) that has been shown to be involved in synergistic activation of ATPase by DnaJ. The overlined residues indicate DnaK amino acids predicted to interact with Mg-ADP. The dashed underline indicates a motif found in DnaK from all gram-negative bacteria which is thought to be essential for ATP-dependent cooperative function with DnaJ and GrpE. The threonine (T) with the dot is required for ATPase activity. (PDF) [file pntd.0007464.s003.pdf]

Fig. S3

```
Wigglesworthia       MGNIIGIDLGTTNSCVAIIENGKVKVIENSEGDRTTPSIIAYTEENEILVGQPAKRQSVT    60
WP_067568929.1       MGKIIGIDLGTTNSCIAIIEGNKPRVIENSEGDRTTPSIIAYTQDGKILVGQPAKRQSVT    60
WP_083172452.1       MGKIIGIDLGTTNSCIAIIEGNKPRVLENSEGDRTTPSIIAYTQDGKILVGQPAKRQSIT    60
WP_014888738.1       MGKIIGIDLGTTNSCVAIIEGNKPRVLENSEGDRTTPSIIAYTQDGEILVGQPAKRQSVT    60
WP_013975497.1       MGKIIGIDLGTTNSCIAIIEGRKPRVLENSEGDRTTPSIIAYTREGEILVGQPAKRQSVT    60
AIN47473.1           MGKIIGIDLGTTNSCIAIVEGTKPRVLENSEGDRTTPSIIAYTQDGEILVGQPAKRQSAT    60
E.coli               MGKIIGIDLGTTNSCVAIMDGTTPRVLENAEGDRTTPSIIAYTQDGETLVGQPAKRQAVT    60
WP_067497883.1       MGRIIGIDLGTTNSCIALIEGSKPRVLENSEGDRTTPSIIAYTQDGEILVGQPAKRQSVT    60
WP_067567978.1       MGKIIGIDLGTTNSCIAIIEGNKPRVLENSEGDRTTPSIIAYTQDGEILVGQPAKRQSVT    60
WP_014888228.1       MGKIIGIDLGTTNSCIAIIEGKKPHVLENSEGDRTTPSIIAYTQDHEILVGQPAKRQSVT    60
WP_067565807.1       MGKIIGIDLGTTNSCIAIIEGSKPRVLENSEGDRTTPSIIAYTQDGEILVGQPAKRQSVT    60
KYP97672.1           MGKIIGIDLGTTNSCIAIIEGSKPHVLENSEGDRTTPSIIAYTQDGEILVGQPAKRQSVT    60
WP_025244843.1       MGKIIGIDLGTTNSCIAIIEGSKPRVLENSEGDRTTPSIIAYTQDGEILVGQPAKRQSVT    60
Sodalis              MGKIIGIDLGTTNSCIAIIEGSKPRVLENSEGDRTTPSIIAYTQDGEILVGQPAKRQSVT    60
WP_074011646.1       MGKIIGIDLGTTNSCIAIIEGSKPRVLENSEGDRTTPSIIAYTQDGEILVGQPAKRQSVT    60
                     **.***********:*:::. . :*:**:*************.: : *********: *

Wigglesworthia       NPKNTFFAIKRLIGRKFTDHEVQRDVNIMPYKIVSSENGDVWLNVKNQKVAPPQISAEIL    120
WP_067568929.1       NPKNTLFAIKRLIGRRFQDDEVQRDVNIMPYKIIAADNGDAWIEVKGQKIAPPQVSAEIL    120
WP_083172452.1       NPKNTLFAIKRLIGRRFQDNEVQRDVNIMPYKIIAADNGDAWIEVKDQKIAPQQISAEIL    120
WP_014888738.1       NPKNTLFAIKRLIGRRYQDQEVQRDVSIMPYTIISADNGDAWLDVKGQKMAPPQISAEIL    120
WP_013975497.1       NPQNTLFAIKRLIGRRFHDAEVQRDVNIMPYKIIAADNGDAWLEVKGQKIAPPQISAEIL    120
AIN47473.1           NPQNTLFAIKRLIGRRFQDEEVQRDVNIMPYKIIAADNGDAWLEIKGQKMAPPQISAEIL    120
E.coli               NPQNTLFAIKRLIGRRFQDEEVQRDVSIMPFKIIAADNGDAWVEVKGQKMAPPQISAEVL    120
WP_067497883.1       NPKNTLFAIKRLIGRRYQDEEVQRDVNIMPYKIIAADNGDAWLEIKGQKIAPPQISAEIL    120
WP_067567978.1       NPKNTLFAIKRLIGRRYQDEEVQRDVNIMPYKIIAAANGDAWLEVKGQKMAPPQISAEIL    120
WP_014888228.1       NPQNTLFAIKRLIGRRFKDEEVQRDVSIMPYKIIEADNGDAWLEVKGQKMAPPQIAAEIL    120
WP_067565807.1       NPKNTLFAIKRLIGRRYQDEEVQRDVSIMPYKIIASDNGDAWLEVKGQKMAPPQISAEIL    120
KYP97672.1           NPQNTLFAIKRLIGRRFKDEEVQRDVSIMPYKIIAADNGDAWLEVKGQKMAPPQISAEIL    120
WP_025244843.1       NPQNTLFAIKRLIGRRFKDEEVQRDVSIMPYKIIAADNGDAWLEVKGQKMAPPQISAEIL    120
Sodalis              NPQNTLFAIKRLIGRRYQDEEVQRDVSIMPYKIVAADNGDAWLEVKGQKMAPPQISAEIL    120
WP_074011646.1       NPQNTLFAIKRLIGRRYPDEEVQRDVSIMPYKIVAADNGDAWLEVKGQKMAPPQISAEIL    120
                     **:**:*********:: * ******.***:.*: : ***.*:::*.**:** *::**:*

Wigglesworthia       KKMKKTAEDYIGKSITEAVITVPAYFNDTQRQATKDAGKIAGLDVKRIINEPTAAALAYG    180
WP_067568929.1       KKMKKTVEDYLGESVTEAVITVPAYFNDAQRQATKDAGRIAGLDVKRIINEPTAAALAYG    180
WP_083172452.1       KKMKKTVEDYLGESVTEAVITVPAYFNDAQRQATKDAGRIAGLDVKRIINEPTAAALAYG    180
WP_014888738.1       KKMKKTAEAYLGESVTEAVVTVPAYFNDTQRQATKDAGRIAGLDVKRIINEPTAAALAYG    180
WP_013975497.1       KKMKKTAEDYLGEQINEAVITVPAYFNDTQRQATKDAGRIAGLDVKRIINEPTAAALAYG    180
AIN47473.1           KKMKKTAEDYLGESITEAVITVPAYFNDTQRQATKDAGRIAGLEVKRIINEPTAAALAYG    180
E.coli               KKMKKTAEDYLGEPVTEAVITVPAYFNDAQRQATKDAGRIAGLEVKRIINEPTAAALAYG    180
WP_067497883.1       KKMKKTAEDYLGESVTEAVITVPAYFNDTQRQATKDAGRIAGLDVKRIINEPTAAALAYG    180
WP_067567978.1       KKMKKTAEDHLGESVTEAVITVPAYFNDTQRQATKDAGRIAGLDVKRIINEPTAAALAYG    180
WP_014888228.1       KKMKKTAEDYLGEPVTEAVITVPAYFNDTQRQATKDAGRIAGLDVKRIINEPTAAALAYG    180
WP_067565807.1       KKMKKTAEDYLGESVTEAVITVPAYFNDTQRQATKDAGRIAGLDVKRIINEPTAAALAYG    180
KYP97672.1           KKMKKTAEDYLGEPVIDAVITVPAYFNDTQRQATKDAGRIAGLDVKRIINEPTAAALAYG    180
WP_025244843.1       KKMKKTAEDYLGEPVTEAVITVPAYFNDTQRQATKDAGRIAGLDVKRIINEPTAAALAYG    180
Sodalis              KKMKKTAEDYLGEPVTEAVITVPAYFNDTQRQATKDAGRIAGLDVKRIINEPTAAALAYG    180
WP_074011646.1       KKMKKTAEDYLGEPVTEAVITVPAYFNDTQRQATKDAGRIAGLDVKRIINEPTAAALAYG    180
                     ******.* ::*: : :**:********:*********:****:***************

                                   ●
Wigglesworthia       LDKKTGNRIIAVYDLGGGTFDISIIEIDDVDGEKTFEVLSTNGDTHLGGEDFDSRLINYL    240
WP_067568929.1       LDKETSNRTIAVYDLGGGTFDISIIEVDDVDGEKTFEVLSTNGDTHLGGEDFDSRLINYL    240
WP_083172452.1       LDKETSNRTIAVYDLGGGTFDISIIEVDDVDGEKTFEVLSTNGDTHLGGEDFDSRLINYL    240
WP_014888738.1       LDKETGNRTIAVYDLGGGTFDISIIEIDDVDGEKTFEVLATNGDTHLGGEDFDSRLINYL    240
WP_013975497.1       LDKETGNRTIAVYDLGGGTFDISIIEIDDVEGEKTFEVLATNGDTHLGGEDFDSRLINYL    240
AIN47473.1           LDKEIGNRTIAVYDLGGGTFDISIIEIDDVDSEKTFEVLATNGDTHLGGEDFDSRLINYL    240
E.coli               LDKGTGNRTIAVYDLGGGTFDISIIEIDEVDGEKTFEVLATNGDTHLGGEDFDSRLINYL    240
WP_067497883.1       LDKETGNRIISVYDLGGGTFDISIIEIDSVEGEKTFEVLATNGDTHLGGEDFDSRLINYL    240
WP_067567978.1       LDKETGNRTIAVYDLGGGTFDISIIEIDDVDGEKTFEVLATNGDTHLGGEDFDSRLINYL    240
WP_014888228.1       LDKETGNRTIAVYDLGGGTFDISIIEIDDVDGEKTFEVLSTNGDTHLGGEDFDSRLINYL    240
WP_067565807.1       LDKETGNRTIAVYDLGGGTFDISIIEIDDVEGEKTFEVLATNGDTHLGGEDFDSRLINYL    240
KYP97672.1           LDKETGNRTIAVYDLGGGTFDISIIEIDDVDGEKTFEVLATNGDTHLGGEDFDSRLINYL    240
WP_025244843.1       LDKETGNRTIAVYDLGGGTFDISIIEIDDVDGEKTFEVLATNGDTHLGGEDFDSRLINYL    240
```

```
Sodalis             LDKETGNRTIAVYDLGGGTFDISIIEIDDVDGEKTFEVLATNGDTHLGGEDFDSRLINYL    240
WP_074011646.1      MDKETGNRTIAVYDLGGGTFDISIIEIDDVDGEKTFEVLATNGDTHLGGEDFDSRLINYM    240
                    :**  .** *:***************:*.*:.*******:*******************:

Wigglesworthia      VNEFKKEQGIDLRNDPLAMQRLKESSEKAKIELSSVHQTDVNLPYITADSSGPKHMNIKV    300
WP_067568929.1      VDEFKKDQGIDLRNDPLAMQRLKEAAEKAKIELSSSQQTDVNLPYITADSFGPKHMNLKV    300
WP_083172452.1      VDEFKKDHGIDLRNDPLAMQRLKEAAEKAKIELSSSQQTDVNLPYITADNFGPKHMNLKV    300
WP_014888738.1      VDEFKKDQGIDLRNDPLAMQRLKETAEKAKIELSSTQQTDVNLPYITADSSGPKHMNIKV    300
WP_013975497.1      VDEFKKDQGIDLRNDPLAMQRLKEAAEKAKIELSSAQQTEVNLPYITADSSGPKHMHLKV    300
AIN47473.1          VDEFKKDQGIDLRNDPLAMQRLKEAAEKAKIELSAAQQTDVNLPYITADATGPKHMNLKV    300
E.coli              VEEFKKDQGIDLRNDPLAMQRLKEAAEKAKIELSSAQQTDVNLPYITADATGPKHMNIKV    300
WP_067497883.1      VDEFKKDQGIDLRNDPLAMQRLKEAAEKAKIELSSAQQTDVNLPYITADGSGPKHMNLKV    300
WP_067567978.1      VDEFKKEQGIDLRNDPLAMQRLKDAAEKAKIELSSAQQTDVNLPYITADATGPKHMNLKV    300
WP_014888228.1      VDAFKKEQGIDLRNDPLAMQRLKEAAEKAKIELSSAQQTDVNLPYITADGAGPKHMNLKV    300
WP_067565807.1      VDEFKKDQGVDLRNDSLAMQRLKEAAEKAKIELSSAQQSDVNLPYITADVSGPKHMNLKV    300
KYP97672.1          VDEFKKDQGIDLRNDPLAMQRLKEAAEKAKIELSSAQQTDVNLPYITADGSGPKHMNLKV    300
WP_025244843.1      VDEFKKDQGIDLRNDPLAMQRLKEAAEKAKIELSSAQQTDVNLPYITADGSGPKHMNLKM    300
Sodalis             VDEFKKDQGIDLRNDPLAMQRLKEAAEKAKIELSSAQQTDVNLPYITADGSGPKHMNLKV    300
WP_074011646.1      VDEFKKDQGIDLRNDPLAMQRLKEAAEKAKIELSSAQQTDVNLPYITADGSGPKHMNLKV    300
                    *: ***::*:***** *******:::********: :*::*********  *****::*:

Wigglesworthia      TRAKLESLVEELIYKTLEPVKTSLKDAKLKIIDIKDVILVGGQTRMPLVQKKVSDFFGKE    360
WP_067568929.1      TRAKLELLVEELVNRTLEPLKLALKDTGLSVSDIKDVILVGGQTRMPLVQRKVTEFFGKE    360
WP_083172452.1      TRAKLELLVEELVNRTLEPLKLALKDTGLSVSDIKDVILVGGQTRMPLVQRKVTEFFGKE    360
WP_014888738.1      TRAKLESLVEDLVNRTLEPLKVALKDASLSVAEIKDVILVGGQTRMPLVQKKVTDFFRKA    360
WP_013975497.1      TRAKLESLVEELVNRTLDPLKVALNDAGLSVIDIKDVILVGGQTRMPLVQKKVTDFFEKE    360
AIN47473.1          TRAKLESLVEDLVHRTMEPLKVALKDAGLSISDIKDVILVGGQTRMPLVQKKVTDFFSKE    360
E.coli              TRAKLESLVEDLVNRSIEPLKVALQDAGLSVSDIDDVILVGGQTRMPMVQKKVAEFFGKE    360
WP_067497883.1      TRAKLESLVEDLVNRTLEPLKVALKDAGLSVSDIKDVILVGGQTRMPLVQKKVTDFFSKE    360
WP_067567978.1      TRAKLESLVEALVNRTLEPLKVALKDASLSVSDIKDVLLVGGQTRMPLVQKKVSDFFGKE    360
WP_014888228.1      TRAKLASLVEELVNRTLAPLEVALKDAGLSVSDIKDVILVGGQTRMPLVQKKVTDFFGKE    360
WP_067565807.1      TRAKLESLVEKLVNRTLEPLKVALKDASLSVSDIKDVILVGGQTRMPLVQKRVTDFFGKE    360
KYP97672.1          TRAKLESLVEELVNRTLEPLKVALKDAGLSVSDIKDVILVGGQTRMPLVQKKVTDFFGKE    360
WP_025244843.1      TRAKLESLVEELVNRTLEPLKVALKDAGLSVSDIKDVILVGGQTRMPLVQKKVTDFFGKE    360
Sodalis             TRAKLESLVEELVNRTLEPLKVALKDAGLSVSDIKDVILVGGQTRMPLVQKKVTDFFGKE    360
WP_074011646.1      TRAKLESLVEELVNRTLEPLKVALKDAGLSVSDIKDVILVGGQTRMPLVQKKVTDFFGKE    360
                    *****  *** *: ::: *:: :*:*: *.: :*.**:*********:**::*::** *

Wigglesworthia      PRKDVNPDEAVAIGAAVQGGVLAGDVKDVLLLDVTPLSLGIETMGGVMTTLISKNTTIPT    420
WP_067568929.1      PRKDVNPDEAVAIGAAIQGGVLAGDVKDVLLLDVTPLSLGIETMGGVMTPLISKNTTIPT    420
WP_083172452.1      PRKDVNPDEAVAIGAAIQGGVLAGDVKDVLLLDVTPLSLGIETMGGIMTQLISKNTTIPT    420
WP_014888738.1      PRKDVNPDEAVAIGAAVQGGVLAGDVKDVLLLDVTPLSLGIETMGGVMTPLITKNTTIPT    420
WP_013975497.1      PRKDVNPDEAVAIGAAVQGGVLTGDVKDVLLLDVTPLSLGIETMGGVMTPLIVKNTTIPT    420
AIN47473.1          PRKDVNPDEAVAIGAAVQGGVLSGNVKDVLLLDVTPLSLGIETMGGVMTLLIAKNTTIPT    420
E.coli              PRKDVNPDEAVAIGAAVQGGVLTGDVKDVLLLDVTPLSLGIETMGGVMTTLIAKNTTIPT    420
WP_067497883.1      PRKDVNPDEAVAIGAAVQGGVLTGNVKDVLLLDVTPLSLGIETMGGVMTPLISKNTTIPT    420
WP_067567978.1      PRKDVNPDEAVAIGAAVQGGVLAGDVKHVLLLDVTPLSLGIETMGGVMTPLIAKNTTIPT    420
WP_014888228.1      PRKDVNPDEAVGIGAAVQGGVLSGDVKDVLLLDVTPLSLGIETMGGVMTPLISKNTTIPT    420
WP_067565807.1      PRKDVNPDEAVAIGAAVQGGVLAGDVKDVLLLDVTPLSLGIETMGGVMTPLIAKNTTIPT    420
KYP97672.1          PRKDVNPDEAVAIGAAVQGGVLAGDVKDVLLLDVTPLSLGIETMGGVMTPLIAKNTTIPT    420
WP_025244843.1      PRKDVNPDEAVAIGAAVQGGVLAGDVKDVLLLDVTPLSLGIETMGGVMTPLIAKNTTIPT    420
Sodalis             PRKDVNPDEAVAIGAAVQGGVLAGDVKDVLLLDVTPLSLGIETMGGVMTPLIAKNTTIPT    420
WP_074011646.1      PRKDVNPDEAVAIGAAVQGGVLAGDVKDVLLLDVTPLSLGIETMGGVMTPLITKNTTIPT    420
                    ***********.****:*****:*:**.******************:** ** *******

Wigglesworthia      KHSQIFSTAEDNQSAVTIHVLQGERKRSIDNKSLGQFNLDGIAPAMRGMPQIEVTFDIDA    480
WP_067568929.1      KHSQVFSTAEDNQSAVTIHVLQGERKRAVDNKSLGQFNLDGIAPAMRGTPQIEVTFDIDA    480
WP_083172452.1      KHSQVFSTAEDNQSAVTIHVLQGERKRAVDNKSLGQFNLDGIAPAMRGTPQIEVTFDIDA    480
WP_014888738.1      KHSQVFSTAEDNQSAVTIHVLQGERKRSSDNKSLGQFNLDGILPAMRGTPQIEVTFDIDA    480
WP_013975497.1      KHSQIFSTAEDNQSAVTIHVLQGERKRAGDNKSLGQFNLDGIAPAIRGTPQIEVTFDIDA    480
AIN47473.1          KHSQVFSTAEDNQSAVTIHVLQGERKRAGDNKSLGQFNLDGITPAMRGMPQIEVTFDIDA    480
E.coli              KHSQVFSTAEDNQSAVTIHVLQGERKRAADNKSLGQFNLDGINPAPRGMPQIEVTFDIDA    480
WP_067497883.1      KHSQVFSTAEDNQSAVTIHVLQGERKRSIDNKSLGQFNLDGISPAMRGIPQIEVTFDIDA    480
WP_067567978.1      KHSQVFSTAEDNQSAVTIHVLQGERKRSCDNKSLGQFNLDGIAPAMRGTPQIEVTFDIDA    480
WP_014888228.1      KHSQVFSTAEDNQSAVTIHVLQGERKRASDNKSLGQFNLDGIAAAMRGKPQIEVTFDIDA    480
WP_067565807.1      KHSQVFSTAEDNQSAVTIHVLQGERKRSGDNKSLGQFNLDGIAPAMRGTPQIEVTFDIDA    480
KYP97672.1          KHSQVFSTAEDNQSAVTIHVLQGERKRAVDNKSLGQFNLDGIAPAMRGTPQIEVTFDIDA    480
```

```
WP_025244843.1      KHSQVFSTAEDNQSAVTIHVLQGERKRTGDNKSLGQFNLDGIAAAMRGTPQIEVTFDIDA    480
Sodalis             KHSQVFSTAEDNQSAVTIHVLQGERKRSGDNKSLGQFNLDGISPAMRGTPQIEVTFDIDA    480
WP_074011646.1      KHSQVFSTAEDNQSAVTIHVLQGERKRSGDNKSLGQFNLDGISPAMRGTPQIEVTFDIDA    480
                    ****:*********************: *************  * ** ***********

Wigglesworthia      DGILHVSAKDKNSGREQKITIKASSGLSENEIDKMLKESEANAELDIKFEELVKTKNQAD    540
WP_067568929.1      DGILHVSAKDKNSGREQKITIKASSGLKEEEIKKMVQEAEANAESDRKFEELVQIRNQAD    540
WP_083172452.1      DGILHVSAKDKNSGREQKITIKASSGLKEEEIQKMVQEAEANAESDRKFEELVQIRNQAD    540
WP_014888738.1      DGILHVSAKDKQSGREQNITIKASSGLNEDEIKKMVKEAEANAVSDRKFEELVQTRNQAD    540
WP_013975497.1      DGILHVSAKDKNSGHEQKITIKASSGLNEAEIKKMVQEAEVNAESDRKFEELVQTRNQAD    540
AIN47473.1          DGILHVSAKDKKSGREQKITIKASSGLSEDEIKKMVQEAEANAESDRKFEELVQTRNQAD    540
E.coli              DGILHVSAKDKNSGKEQKITIKASSGLNEDEIQKMVRDAEANAEADRKFEELVQTRNQGD    540
WP_067497883.1      DGILHVSAKDKKNGREQKITIKASSGLNEEEIQKMVKEAEANIESDRKFEELVQVRNQAD    540
WP_067567978.1      DGILHVSAKDKNSGREQKITIKASSGLNEQEIQKMVLDAEANAESDRKFEELVQTRNQAD    540
WP_014888228.1      DGILHVSAKDKNSGREQKITIKASSGLNEEEIQKMVQEAEANAESDRKFEELVQTRNQAD    540
WP_067565807.1      DGILHVSAKDKNSGREQKITIKASSGLSEKEIQKMVQEAEANAEFDRKFEDLVQTRNRAD    540
KYP97672.1          DGILHVSAKDKNSGREQKITIKASSGLNEEEIQKMVQEAEANAESDRKFEDLVRTRNQAD    540
WP_025244843.1      DGILHVSAKDKNSGREQKITIKASSGLNEEEIQKMVQEAEANAESDRKFEELVQTRNQAD    540
Sodalis             DGILHVSAKDKNSGREQKITIKASSGLNEEEIQKMVQEAEANAESDRKFEALVQTRNQAD    540
WP_074011646.1      DGILHVSAKDKNSGREQKITIKASSGLNEEEIQKMVQEAEANAESDRKFEELVQTRNQAD    540
                    ***********:.*:**:*********.* **.**: ::*.*   * *** **: :*:.*

Wigglesworthia      HLLHSTRKQIKEA--KNLPLEKKTEIEKCINELELSIKGEDKKDIEIKIQSLIQISSCLV    598
WP_067568929.1      HLLHSTRKQLKES-SDKLLVNDKEAIEEALKNLDTSLKGEDKSDIEAKIQTVIQLSSKLI    599
WP_083172452.1      HLLHSTRKQLKEY-GAKLLENDKLAIEEALKNLDTSLKGEVKSDIENKIQLLIKVSSKLI    599
WP_014888738.1      HILHSTRKQLEDI-GDKLSSENKAAVENAIKNLHIALKDENKSDIETKMQDLVQVSAKLL    599
WP_013975497.1      YMLHSTRKQLEDV-GEKLSTEVKTPIEVALKDLEAVLKEEDKAKIDEKMQNLIQVSSQLL    599
AIN47473.1          NLMHSTSKQLEEH-SNKLSNEDKNQIEDAINNLNIALKDESKADIETKMQALIQVSSKCL    599
E.coli              HLLHSTRKQVEEA-GDKLPADDKTAIESALTALETALKGEDKAAIEAKMQELAQVSQKLM    599
WP_067497883.1      HLLHSTRKQLEDT-TDKLSSDDKIAIENALKDLDTAMKSEDKTDIEAKMQTLIQVSEKLL    599
WP_067567978.1      HLLHSTRKQLEDA-GNKLPDNDKTAIAEALKDLDTSLKSEDKANIEAKMKVLIQLSGKLL    599
WP_014888228.1      HLLHSTRKQLEDM-CGTLPSNDKAAIEEALHDLESSLKGTDKADIEAKMQGLIQLSGKLL    599
WP_067565807.1      HLMHSTRKQLSEVEADKLPTDDKTAIEDALKNLDTALKGEDKADIETKIQVLIQVSGKLF    600
KYP97672.1          HLLHSTRKQLED-AGDKLPAGDKNVIEDVLKNLDTALKGEDKADIEAKMQALIQVSGKLL    599
WP_025244843.1      HLLHSTRKQLED-AGDKLPADDKSAIEEALKNLDTALKGEDKADIEAKMQALIQVSGKLL    599
Sodalis             HLLHSTRKQLED-AGDKLPADDKTAIEDALKNLDTVLKGEDKADIEAKMQALIQVSGKLL    599
WP_074011646.1      HLLHSTRKQLED-AGDKLPAEDKTAIEDALKNLDTALKGEDKADIEAKMQALIQVSGKLL    599
                     ::*** **:.:    .*    *  :   :  *.  :*   *  *: *:: : ::*   .

Wigglesworthia      DFSKNKENLNKEDIIKTNKKENNNKTNDDVVDAEFEEIKDKKN   641
WP_067568929.1      EVAQQK--DKDE--VKGSGDN-NSKAENDVVDAEFEEVKDKK-   636
WP_083172452.1      EVAQHQKNEKDE--VKESGDNNNAKTENEVVDAEFEEVKDKK-   639
WP_014888738.1      VEESPKKDQQVY--E-Q-STTGGNAKDDDVVDAEFEEVKDKK-   637
WP_013975497.1      EAVQHKDNT---------VEGSSNKVDSDVVDAEFEEVKDKK-   632
AIN47473.1          KFTQQRTQAAD-------DNNDNTKIDDDVVDAEFEEVKDKK-   634
E.coli              EIAQQQHAQQQT--A-GADASANNAKDDDVVDAEFEEVKDKK-   638
WP_067497883.1      EAAKQQAQAASK--S-N-TEDVSAKASDDVVDAEFEEVKDKK-   637
WP_067567978.1      EVVQQQAQMARE--D-N-VDGGTKATSEDVVDADFEEVKDKK-   637
WP_014888228.1      SAAQQHAQAGSA--G-G-TDHC-IKPEEDVVDAEFEEIKDKK-   636
WP_067565807.1      EAAQQKARSASE--S-S-ANGS-AKVDDDVVDAEFEEVKDKK-   637
KYP97672.1          KVAQQQAQAAGE--G-G-TDGG-AKADDDVVDAEFEEVKDKK-   636
WP_025244843.1      EVAQQQAQAAGE--G-G-T-------DDDVVDAEFEEVKDKK-   630
Sodalis             EVAQQQAQAAGD--G-G-ADGS-AKADDDVVDAEFEEVKDKK-   636
WP_074011646.1      EVAQQQAQAAGE--G-G-ADGS-AKADDDVVDAEFEEVKDKK-   636
                       . :                     ..:****:***:****
```
