# Supplementary material for: Thermal stress responses of Sodalis glossinidius, an indigenous bacterial symbiont of hematophagous tsetse flies
Source: PLoS Negl Trop Dis. 2019 Nov 18;13(11):e0007464. doi: 10.1371/journal.pntd.0007464 (PMC6887450; doi:10.1371/journal.pntd.0007464)
Supplement: S1 Table — (DOCX) [file pntd.0007464.s004.docx]

**Table S1. Bacterial strains and plasmids.**

| Strain or Plasmid | Characteristics | Reference |
| --- | --- | --- |
|  |  |  |
| Bacterial strains |  |  |
| *E. coli* strains |  |  |
| DH5α | *endA1 hsdR17 supE44 thi-1 recA1 gyrA relA1* Δ(*lacZYA*-*argF*)*U169* *deoR* [Φ80*dlac*Δ(*lacZ*)*M15*] | [89] |
| NEB 5-alpha | fhuA2 Δ(argF-lacZ)U169 phoA glnV44 Φ80 Δ(lacZ)M15 gyrA96 recA1 relA1 endA1 thi-1 hsdR17 | New England Biolabs |
| MC4100 | F-, *araD139,* Δ*(argF-lac*)U169, *rpsL150*, *relA1*, *deoC1*, *ptsF25*, *rpsR*, *ftbB301* | [90] |
| MC4100ΔdnaK | F-, araD139*,* Δ*(argF-lac*)U169, *rpsL150*, *relA1*, *deoC1*, *ptsF25*, *rpsR*, *ftbB301*, Δ*dnaK52::cam* | [91] |
| BW25113 | F-, Δ*(araD-araB)567*, Δ*lacZ4787*(::rrnB-3), λ-, *rph-1*, Δ*(rhaD-rhaB)568*, *hsdR514* | [92,93] |
| JW0014-1 | F-, Δ*(araD-araB)567*, Δ*lacZ4787*(::rrnB-3), λ-, *rph-1*, Δ*(rhaD-rhaB)568*, *hsdR514*, Δ*dnaJ735*::*kan* | [92] |
| DA15 | F-, *galT22*, λ-, *pheA18::Tn10*, *IN(rrnD-rrnE)1*, *rph-1* | [94] |
| DA16 | F-, *galT22*, λ-, *pheA18::Tn10*, *IN(rrnD-rrnE)1*, *rph-1*, *grpE280* | [94] |
|  |  |  |
|  |  |  |
| *Sodalis* strains |  |  |
| SOD^F^ | *S. glossindius* from *Glossina moristans moristans* | B. Weiss |
|  |  |  |
| Plasmids |  |  |
| pWKS30 | Low-copy-number cloning vector; carb^R^ | [95] |
| pJR1 | *Sodalis dnaK* in pWKS30; carb^R^ | This study |
| pJR5 | *Sodalis dnaK* and *dnaJ* in pWKS30; carb^R^ | This study |
| pJS2 | *E. coli dnaK* gene in pWKS30; carb^R^ | This study |
| pSD2 | *Sodalis dnaJ* gene in pWKS30; carb^R^ | This study |
| pRF2 | *Sodalis grpE* in pWKS30; carb^R^ | This study |
