# Supplementary material for: Thermal stress responses of Sodalis glossinidius, an indigenous bacterial symbiont of hematophagous tsetse flies
Source: PLoS Negl Trop Dis. 2019 Nov 18;13(11):e0007464. doi: 10.1371/journal.pntd.0007464 (PMC6887450; doi:10.1371/journal.pntd.0007464)
Supplement: S2 Table — (DOCX) [file pntd.0007464.s005.docx]

**Table S2. PCR primers used in this study.**

| **Primer pairs** | **Sequence** | **DNA amplified** |
| --- | --- | --- |
|  |  |  |
| UR423 | 5’CTAGTCTAGACGCAACTGTTCCTGACCTAAG | *Sodalis dnaK* |
| UR424 | 5’CCCGCTCGAGAAAATATCGCCAAACACATCG |  |
|  |  |  |
| UR455 | 5’GGCTCTAGATAGCGGGCGATCTGATGTTC | *Sodalis* *grpE* |
| UR456 | 5’AAAACTGCAGGGCCAACAGCCCATTGATTG |  |
|  |  |  |
| UR518 | GCTCTAGAGGGAAGAGGTAGGGGGATGA | *E. coli dnaK* |
| UR519 | CCGCTCGAGGCGAGTCGGTCAGAACTTCA |  |
|  |  |  |
| UR530 | CGATATCTTTGGCGATGTGTTTGGCGATATTTTTGGCGGCGGCCGGCG | *Sodalis dnaJ* |
| UR531 | GTACCGGGCCCCCCCTCGAGGCGGTAGGGGTAAATGAAAACCG |  |
|  |  |  |
| UR545 | CCAGGATGAAGAAGTACAGC | *Sodalis dnaK* internal |
| UR546 | GTTCGCCCAGATAGTCTTC | for qPCR |
|  |  |  |
| UR556 | GGTCGGGTAGAGAAATCCAAG | *Sodalis dnaJ* internal |
| UR557 | AGAGTTTACCGGTCTGCGTTT | for qPCR |
|  |  |  |
| UR558 | ATGAGCGTATTGCTGAATTGG | *Sodalis grpE* internal |
| UR559 | CCTTCTCGACATCCTGTTCAC | for qPCR |
|  |  |  |
| QrplB1F | TTCAGATCGTGGCTCGTGATGG | *Sodalis rplB* internal |
| QrplB1R | GCAGCATGTGTTCGGCATTACC | for qPCR |
|  |  |  |
|  |  |  |
