# Supplementary material for: Thermal stress responses of Sodalis glossinidius, an indigenous bacterial symbiont of hematophagous tsetse flies
Source: PLoS Negl Trop Dis. 2019 Nov 18;13(11):e0007464. doi: 10.1371/journal.pntd.0007464 (PMC6887450; doi:10.1371/journal.pntd.0007464)
Supplement: S3 Table — (DOCX) [file pntd.0007464.s006.docx]

**Table S3. *Sodalis* DnaK homology with other insect symbionts.**

| **Bacterial species** | **Host** | **Host common name** | **% identity^a^** | **% similarity^a^** |
| --- | --- | --- | --- | --- |
| *Candidatus Sodalis sp. SoCistrobi* | *Cinara strobi* | White pine aphid | 99 | 99 |
| *Sodalis-*like endosymbiont of *Proechinophthirus fluctus* | *Proechinophthirus fluctus* | Seal louse | 97 | 98 |
| *Candidatus Sodalis pierantonius str. SOPE* | *Sitophilus oryzae* | Rice weevil | 97 | 98 |
| *Candidatus Doolittlea endobia* ^b^ | *Maconellicoccus hirsutus** | Hibiscus mealy bug | 94 | 97 |
| *Candidatus Hoaglandella endobia* ^b^ | *Trionymus perrisii** | Mealy bug | 93 | 96 |
| Secondary endosymbiont of *Ctenarytaina eucalypti* | *Ctenarytaina eucalypti* | Blue gum psyllid | 92 | 95 |
| Candidatus *Gullanella endobia* ^b^ | *Ferrisia virgata** | Striped mealy bug | 92 | 95 |
| *Candidatus Mikella endobia* ^b^ | *Paracoccus marginatus** | Papaya mealy bug | 90 | 96 |
| *Candidatus Baumannia cicadellinicola BGSS* | *Homalodisca coagulata* | Glassy-winged sharpshooter | 89 | 94 |
| Secondary endosymbiont of *Heteropsylla cubana* | *Heteropsylla cubana* | Jumping plant lice | 89 | 94 |
| Secondary endosymbiont of *Trabutina mannipara* | *Trabutina mannipara* | Mealy bug | 88 | 94 |
| *Candidatus Moranella endobia* ^b^ | *Planococcus citri** | Citrus mealy bug | 88 | 94 |
| *Wigglesworthia glossinidia morsitans* | *Glossina morsitans* | tsetse fly | 81 | 90 |

^a^Percent identity and similarity were calculated from BLAST searches of *Sodalis* DnaK with the microbial databases at the National Center for Biotechnology Information (www.ncbi.nlm.nih.gov) and at Kyoto Encyclopedia of Genes and Genomes ([www.kegg.jp](http://www.kegg.jp))

^b^The indicated bacterial species are intra-bacterial symbionts of the β-proteobacterium *Tremblaya princeps*, which resides within the indicated insect host [96].
