# Supplementary material for: Thermal stress responses of Sodalis glossinidius, an indigenous bacterial symbiont of hematophagous tsetse flies
Source: PLoS Negl Trop Dis. 2019 Nov 18;13(11):e0007464. doi: 10.1371/journal.pntd.0007464 (PMC6887450; doi:10.1371/journal.pntd.0007464)
Supplement: S4 Table — (DOCX) [file pntd.0007464.s007.docx]

**Table S4. *Sodalis* chaperone genes facilitate elevated temperature survival in *E. coli*.**

|  | Growth temperature^a^ | |
| --- | --- | --- |
| Strain | 30 ^o^C | 45+1^o^C |
| MC4100/pWKS30 | + | + |
| MC4100/pJR1 (dnaK^Sod^) | + | + |
| MC4100ΔdnaK/pWKS30 | + | - |
| MC4100ΔdnaK/pJR1 (dnaK^Sod^) | + | + |
| BW25113 /pWKS30 | + | + |
| BW25113 /pSD2 (dnaJ^Sod^) | + | + |
| JW0014-1 (*ΔdnaJ*)/pWKS30 | + | - |
| JW0014-1 (*ΔdnaJ*)/pSD2 (dnaJ^Sod^) | + | + |
| DA15/pWKS30 | + | + |
| DA15/pRF2 (grpE^Sod^) | + | + |
| DA16(*grpE280*)/pWKS30 | + | - |
| DA16(*grpE280*)/pRF2 (grpE^Sod^) | + | + |

^a^Overnight cultures of the indicated *E. coli* strains grown at 30°C were plated on L agar plates

and incubated at the indicated temperatures. +, growth after 24 hours; -, no growth after 24

hours.
