# Supplementary material for: Thermal stress responses of Sodalis glossinidius, an indigenous bacterial symbiont of hematophagous tsetse flies
Source: PLoS Negl Trop Dis. 2019 Nov 18;13(11):e0007464. doi: 10.1371/journal.pntd.0007464 (PMC6887450; doi:10.1371/journal.pntd.0007464)
Supplement: S1 References — (DOCX) [file pntd.0007464.s008.docx]

89. Sambrook J, Fritsch EF, Maniatis T. 1989. Molecular cloning: a laboratory manual, 2nd ed. Cold 533 Spring Harbor Laboratory Press, Cold Spring Harbor, N.Y.

90. Casadaban MJ. Transposition and fusion of the lac genes to selected promoters

in Escherichia coli using bacteriophage lambda and Mu. J Mol Biol. 1976 Jul

5;104(3):541-55. PubMed PMID: 781293.

91. Bukau B, Walker GC. Cellular defects caused by deletion of the Escherichia

coli dnaK gene indicate roles for heat shock protein in normal metabolism. J

Bacteriol. 1989 May;171(5):2337-46. PubMed PMID: 2651398; PubMed Central PMCID:

PMC209906.

92. Baba T, Ara T, Hasegawa M, Takai Y, Okumura Y, Baba M, Datsenko KA, Tomita M,

Wanner BL, Mori H. Construction of Escherichia coli K-12 in-frame, single-gene

knockout mutants: the Keio collection. Mol Syst Biol. 2006;2:2006.0008. Epub 2006

Feb 21. PubMed PMID: 16738554; PubMed Central PMCID: PMC1681482.

93. Datsenko KA, Wanner BL. One-step inactivation of chromosomal genes in

Escherichia coli K-12 using PCR products. Proc Natl Acad Sci U S A. 2000 Jun

6;97(12):6640-5. PubMed PMID: 10829079; PubMed Central PMCID: PMC18686.

 94. Johnson C, Chandrasekhar GN, Georgopoulos C. Escherichia coli DnaK and GrpE

heat shock proteins interact both in vivo and in vitro. J Bacteriol. 1989

Mar;171(3):1590-6. PubMed PMID: 2522091; PubMed Central PMCID: PMC209785.

95. Wang RF, Kushner SR. Construction of versatile low-copy-number vectors for

cloning, sequencing and gene expression in Escherichia coli. Gene. 1991

Apr;100:195-9. PubMed PMID: 2055470.

96. Husnik F, McCutcheon JP. Repeated replacement of an intrabacterial symbiont in

the tripartite nested mealybug symbiosis. Proc Natl Acad Sci U S A. 2016 Sep

13;113(37):E5416-24. doi: 10.1073/pnas.1603910113. Epub 2016 Aug 29. PubMed PMID:

27573819; PubMed Central PMCID: PMC5027413.
